# Effects of new motorway infrastructure on active travel in the local population: a retrospective repeat cross-sectional study in Glasgow, Scotland

**DOI:** 10.1186/s12966-016-0403-9

**Published:** 2016-07-07

**Authors:** Jonathan R. Olsen, Richard Mitchell, David Ogilvie, on behalf of the M74 study team

**Affiliations:** Centre for Research on Environment, Society and Health, Institute of Health and Wellbeing, University of Glasgow, 1 Lilybank Gardens, Glasgow, G12 8RZ UK; MRC Epidemiology Unit & UKCRC Centre for Diet and Activity Research (CEDAR), School of Clinical Medicine, University of Cambridge, Cambridge Biomedical Campus, Box 285, Cambridge, CB2 0QQ UK

**Keywords:** Natural experiment, Active travel, Physical activity, Evaluation

## Abstract

**Background:**

Promoting active travel is an important part of increasing population physical activity, which has both physical and mental health benefits. A key benefit described by the then Scottish Government of the five-mile M74 motorway extension, which opened during June 2011 in the south of Glasgow, was that the forecast reduction in motor traffic on local streets would make these streets safer for walking and cycling, thus increasing active travel by the local population. The aim of the study was to evaluate the impact of new motorway infrastructure on the proportion of journey stages made actively (cycling or on foot) by individuals travelling in and out of the local area.

**Methods:**

Data for the periods 2009–10 and 2012–13 were extracted from the Scottish Household Survey (SHS) travel diaries, which record each journey stage made during the previous day by a representative sample of the Scottish population aged 16 and over. Each individual journey stage was assigned to one of the following study areas surrounding existing and new transport infrastructure: (1) an area surrounding the new M74 motorway extension (*n* = 435 (2009–10), 543 (2012–13)), (2) a comparator area surrounding an existing motorway (*n* = 477 (2009–10), 560 (2012–13)), and (3) a control area containing no comparable motorway infrastructure (*n* = 541 (2009–10), 593 (2012–13)). Multivariable, multi-level regression analysis was performed to determine any between-area differences in change in active travel over time, which might indicate an intervention effect. Reference populations were defined using two alternative definitions, (1) Glasgow City and (2) Glasgow and surrounding local authorities.

**Results:**

The results showed an increase in the proportion of journey stages using active travel in all study areas compared to both reference populations. However, there were no significant between-area differences to suggest an effect attributable the M74 motorway extension.

**Conclusions:**

There was no clear evidence that the M74 motorway extension either increased or decreased active travel in the local area. The anticipation by policy makers that reduced motorised traffic on local streets might increase journeys walked or cycled appears to have been unfounded.

**Electronic supplementary material:**

The online version of this article (doi:10.1186/s12966-016-0403-9) contains supplementary material, which is available to authorized users.

## Background

Increasing the proportion of journeys which are walked or cycled (‘active travel’) is an important component to increasing population level physical activity, which can translate into physical and mental health benefits of significant magnitude [1, 2]. A recent review described the potential effect of increased walking and cycling in urban England and Wales on the National Health Service (NHS) could lead to a saving of approximately £17billion through the reduced prevalence of diseases associated with physical inactivity, if the combination of impacts found in local area studies were immediately realised across this area and maintained for a 20 year period [3]. Besides the health benefits of increasing the number of active journeys, walking or cycling as opposed to motorised transport, is a sustainable transport mode which has environmental benefits in terms of reducing population carbon footprint and air pollution in urban cities [4].

There is some evidence of a beneficial relationship between the built environment and physical activity [5]. ‘*Physical Activity and The Environment’* guidance published by the UK National Institute for Health and Clinical Excellence (NICE) in 2008 [6], and updated during 2014 [7], recommended that when developing or maintaining streets and roads, that pedestrians, cyclists, and other transport modes involving physical activity should be given the highest priority. In the UK the prioritisation of pedestrians and cyclists in urban areas is patchy, partially due to the dominance of motor vehicles, and society’s dependence upon a transport network designed for cars [8]. However, constructing more ‘active’ environments alone may not ultimately be enough to produce increases in active travel levels, particularly for commuter journeys [9] and in deprived urban populations [10]. Therefore it is important that new infrastructure designs are supported by evidence to ensure effectiveness.

In June 2011, a new 5 mile motorway extension, the M74 extension, opened in the South of Glasgow, UK; a city of 599,650 residents [11]. The extension, which is mainly raised above existing roads and dwellings, cost approximately £800 million and crosses a largely urban residential area. An independent local public inquiry in 2003 considered the arguments for and against construction and concluded that the claimed benefits were likely to be ‘ephemeral’, that the new motorway ‘would be very likely to have very serious undesirable results’ for local communities, and therefore recommended against the proposal [12]. Nevertheless, the construction went ahead. One of the key strategic and economic objectives for the construction of the motorway extension was to relieve congestion on local streets and allow priority for public transport, cyclists and pedestrians [13]. Negative impacts of the motorway were anticipated by the Scottish Government, such as undesirable disruption for cyclists and pedestrians on the main feeder lanes to motorway junctions due to increased traffic [12]. The project contained no specific investment in new cycling and walking infrastructure other than artwork and some feature lighting under new and existing M74 bridges [13], but there was other on-going citywide investment during the study period. Other significant investments in the city were linked to the hosting of the 2014 Commonwealth Games, regeneration schemes in the South of the city (such as the Clyde Gateway), and on-going investments by Glasgow City Council and cycling charities. However, quantifying specific impacts of these infrastructure changes for the area surrounding the M74 extension is problematic, highlighting a limitation of natural experiments of this kind.

The aim of this study was to evaluate the effect of the M74 extension on the proportion of journeys stages using active travel (cycling or by foot) for local residents living adjacent to it. The main objectives of the study were:

1. Evaluation of the impact of the M74 extension on changes in the proportion of active journey stages (cycling or by foot) over time.
2. Compare changes in active journey stage by individuals travelling in and out of the intervention area (surrounding the M74 extension) with change in a comparator and control area.

## Methods

### Survey data

Travel diary data were obtained for the complete Scottish Household Survey (SHS); the SHS is a nationally representative rolling cross-sectional survey conducted with adults aged 16+ selected from a cluster-random sample of households in Scotland [14]. Face to face interviews were conducted to obtain socio-demographic data and the participant self-completed a travel diary which details all journey stages completed during the previous day. Data collected include start, end, purpose, distance, and mode of travel for each stage. Journey distances were calculated using straight-line distance between stage start and end point by Transport Scotland (http://www.transportscotland.gov.uk/system/files/uploaded_content/documents/research/Distance_in_the_Travel_Diary.pdf).

Travel diary data were provided by the SHS for the period 2009 to 2013 for all areas of Scotland and aggregated to the stage start, stage end, and participant residential Scottish Intermediate Zone. This is a geographical polygon area based on home address of each participant (Groups of approximately 4000 household residents which respect physical boundaries and natural communities, have a regular shape and contain households with similar social characteristics [15]).

### Active travel

Each travel diary is divided into individual journeys phases to represent different ‘stages’ of a journey (i.e. walk to bus stop, travel on bus, and walk to destination describes three journey stages). For the purpose of our analysis, active travel was defined as a stage that was either walked or cycled; all stages were included regardless of the purpose of the journey and ‘active travel’ was an attribute assigned to each stage of each journey. A stage is assigned to a study area if either the origin or destination is within its boundary. The SHS dataset report only the following two active transport modes: walking or cycling. This is a limitation of the SHS dataset.

### Design and study area definition

**Fig. 1:**
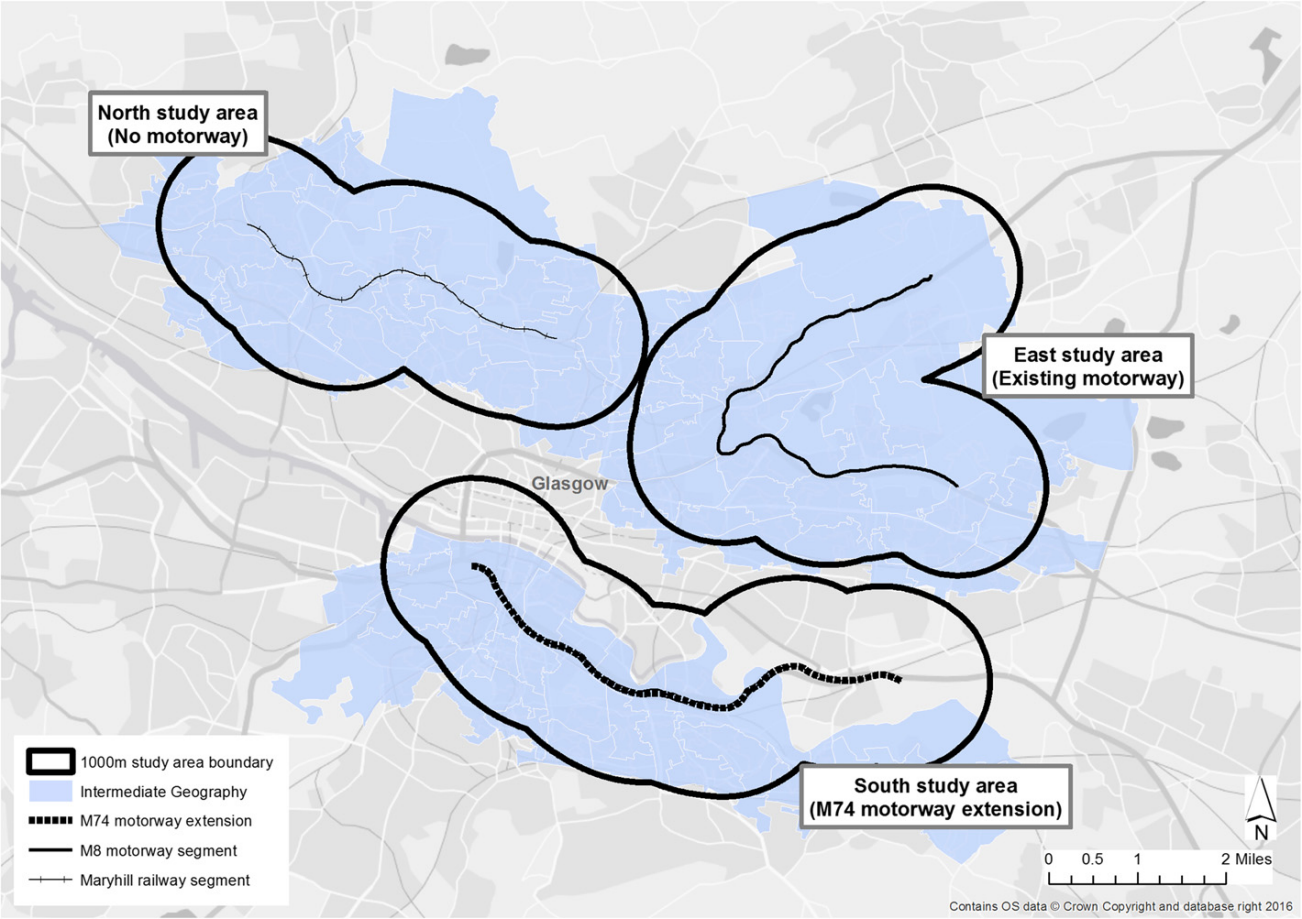
M74 study areas, Glasgow, UK

This study was a component of a larger evaluation which included assessment of impact on road traffic injuries, community perceptions and wellbeing [16]. This design included the identification and use of three study areas; the local area surrounding the new motorway in the *South* of the city (intervention area), a residential area surrounding an existing motorway (the M8) in the *East* of the city (comparator area), and a residential area without a motorway in the *North* of the city (control area). The study areas will be referred to as *North, East* and *South* hereafter. A 1000 metre (0.6 mile) buffer was created around each of these linear transport structures to define the three study areas and Scottish Intermediate Zones either fully or mostly contained within this area were assigned to each study area (Fig. 1); for the *South* study area the River Clyde was used as a natural edge. For this study, changes in active travel over time were compared between these three study areas.

We selected a much larger reference area to provide an indicator of broader secular changes in travel behaviours; the area covered by Glasgow City Council and surrounding local authorities. The area was selected due to the intervention area crossing more than one local administrative authority, and also because it provided a mix of both rural and urban geographies, and road networks for comparison.

To compare active travel, pre- and post-intervention, data were pooled for the period 2009/10 (pre-intervention) and 2012/13 (post-intervention). Although data were also received for 2011, the month of interview was not recorded within the survey dataset and it was not possible to assign these responses to either the ‘pre’ or the ‘post’ condition.

### Statistical analysis

#### Descriptive statistics

Summary statistics described population characteristics in terms of age, gender, self-reported health status and employment. The proportion of stages that were made by active modes was described for all populations. Descriptive statistics provided  for both the *people* sampled, and for all journey stages.

### Multivariable, multi-level models

#### Changes in active travel over time

Logistic regression models were fitted to examine active travel, residential location of participant in terms of study area, and time period (pre or post intervention). The stage was taken as the unit of analysis, and the analysis modelled the likelihood of a stage being ‘active’ by regressing the binary outcome variable for each stage (active yes/no) on the explanatory variables including area and time (and later including the interaction of area and time-period). Models were firstly performed without covariates and then adjusted for age, gender, health status and employment; due to the small numbers of stages by study areas these covariates were dichotomised (a description of original and dichotomised variables are included in Additional file 1: Table S2). Models take account of clustering of journey stages within individuals.

Individuals were weighted to correct for differences in selection probabilities between areas of Scotland, the number of adults in different sized households, and days on which people were available for interview. Analysis was performed weighted and un-weighted with no substantial differences in the main outcomes or messages or the results, however weighting provided a better representation of the 16 to 24 age group when compared to the 2011 Scottish census for the same area; therefore all analyses presented in the manuscript were weighted.

#### Comparing changes in active travel over time between areas

Interactions formally assess between area differences in change over time in terms of odds ratios (OR) for active travel from the baseline reference category of Glasgow and surrounding authorities at the pre-motorway time-period (2009–10). Differences in change between areas may suggest an intervention effect. Predictive margins of active travel are described for the pre and post intervention time period and differences between the predictors of active travel reported. This approach was used for the interaction using the margins commands and accounts for the different distribution of confounders in each group. Significance is assumed at the 5 % level. Analysis was performed in STATA/SE 14.0.

### Sensitivity analysis (reference population and journey distance)

Two sensitivity analyses were performed (Additional file 2: Table S1).

Firstly, we examined the effect of using an alternative and smaller reference boundary in the multivariable analysis. This definition comprises Glasgow City only and excludes the surrounding Local Authorities used in the main analysis (North Lanarkshire, South Lanarkshire, East Renfrewshire, Renfrewshire, West Dunbartonshire and East Dunbartonshire).

The purpose of the second sensitivity analysis is to assess the extents to which including or excluding journey stages that were either certain or unlikely to be 'active' impacted on our results. For example, a journey over 6 km in distance is likely to be made using a motorised transport mode. We examined the effect of assessing journey stages for which there may reasonably have been a ‘choice’ of mode. This used the following four definitions: ***[a]****All stages:* All stages regardless of distance travelled. ***[b]****All stages greater than 0.5 km (kilometre)*: Stages so short a distance that it is reasonable that they could only be walked or cycled were excluded. ***[c]****Stages less than 5 km only*: Stages that were of such a long distance that they are likely only to be walked or cycled by enthusiasts were excluded. And ***[d]****Stages greater than 0.5 km and less than 5 km:* Excluded stages due to both definitions described in *[b]* and *[c]* above.

## Results

### Participant characteristics

During the period 2009–10, 3706 participants in Glasgow and its surrounding authorities completed a SHS travel diary; 43.9 % (n(number):1627) males (Table 1). A greater number of travel diaries were completed during the latter time 2012–13 period (n:4205). Similar proportions of surveys were completed by males and females in each of the study areas for both time periods and no changes in the proportion of diaries completed by either gender were seen by either study area or time period. The majority of participants were aged 25 to 59 (2009–10: 60.7 % and 2012–13: 60.0 %), providing a representative population (compared to 2011 Scottish Census data) [17]. Weighted populations of the 16 to 24 age group show differences between 0.2 and 1.2 % by study area compared to 2011 Scottish Census.

**Table 1:** Study population demographics

Demographics		2009–10				2012–13			
		Glasgow and surrounding authorities	Study Area 1 (South)	Study Area 2 (East)	Study Area 3 (North)	Glasgow and surrounding authorities	Study Area 1 (South)	Study Area 2 (East)	Study Area 3 (North)
		*N (%)*	*N (%)*	*N (%)*	*N (%)*	*N (%)*	*N (%)*	*N (%)*	*N (%)*
Age									
	16 to 24	328 (8.9)	14 (7.6)	16 (8.1)	21 (10.7)	373 (8.9)	12 (6.1)	20 (10.5)	13 (6.1)
	25 to 59	2251 (60.7)	121 (65.4)	122 (61.9)	120 (61.2)	2524 (60.0)	140 (71.1)	113 (59.2)	139 (65.0)
	60 plus	1127 (30.4)	50 (27.0)	59 (29.9)	55 (28.1)	1308 (31.1)	45 (22.8)	58 (30.4)	62 (29.0)
Gender									
	Male	1627 (43.9)	79 (42.7)	76 (38.6)	88 (44.9)	1874 (44.6)	92 (46.7)	79 (41.4)	100 (46.7)
	Female	2079 (56.1)	106 (57.3)	121 (61.4)	108 (55.1)	2331 (55.4)	105 (53.3)	112 (58.6)	114 (53.3)
Current economic status									
	Employed/Education/Training	2079 (56.1)	106 (57.3)	109 (55.3)	111 (56.6)	2399 (57.1)	128 (65.0)	100 (52.4)	124 (57.9)
	Unemployed, seeking work or Unable to work due to sickness	437 (11.8)	19 (10.3)	25 (12.7)	22 (11.2)	475 (11.3)	23 (11.7)	32 (16.8)	28 (13.1)
	Retired	962 (26.0)	43 (23.2)	49 (24.9)	47 (24.0)	1103 (26.2)	37 (18.8)	48 (25.1)	53 (24.8)
	Other	228 (6.2)	17 (9.2)	14 (7.1)	16 (8.2)	228 (5.4)	9 (4.6)	11 (5.8)	9 (4.2)
How is your health in general?									
	Very good/good	2632 (71.2)	125 (67.9)	137 (69.5)	135 (68.9)	3054 (72.6)	154 (78.2)	117 (61.3)	156 (72.9)
	Fair/Bad/Very bad	1066 (28.8)	59 (32.1)	60 (30.5)	61 (31.1)	1151 (27.4)	43 (21.8)	74 (38.7)	58 (27.1)
Number of journey stages (completed on single day of survey) by study area									
	Stages	9777	435	477	541	11684	543	560	593
	Mean (range) number of stages	2.1 (1 to 14)	1.9 (1 to 8)	2.0 (1 to 8)	2.2 (1 to 10)	2.2 (1 to 10)	2.2 (1 to 10)	2.3 (1 to 9)	2.1 (1 to 10)

Individual characteristics of population, individuals may make more than one journey

Over half of participants were either employed, in education or training (2009–10: 56.1 % and 2012–13: 57.1 %), and a substantial proportion were retired (2009–10: 25.9 % and 2012–13: 26.2 %); reflecting the older demographic of the sample. Most of the population described their health as fair to very good (2009–10: 93.3 % and 2012–13: 91.5 %).

The numbers of journey stages completed by individuals during 2009–10 and 2012–13 are presented in Table 1; the mean number of stages completed during the previous day was 2.1 and ranged between 1 to 14.

### Changes in active travel over time

#### Journey stages using active travel

The total number of stages included in 2009–10 by study area were South (n:435), East (n:477), and North (n:541). There were more stages included for 2012–13: South (n:543), East (n: 560), and North (n:593).

**Fig. 2:**
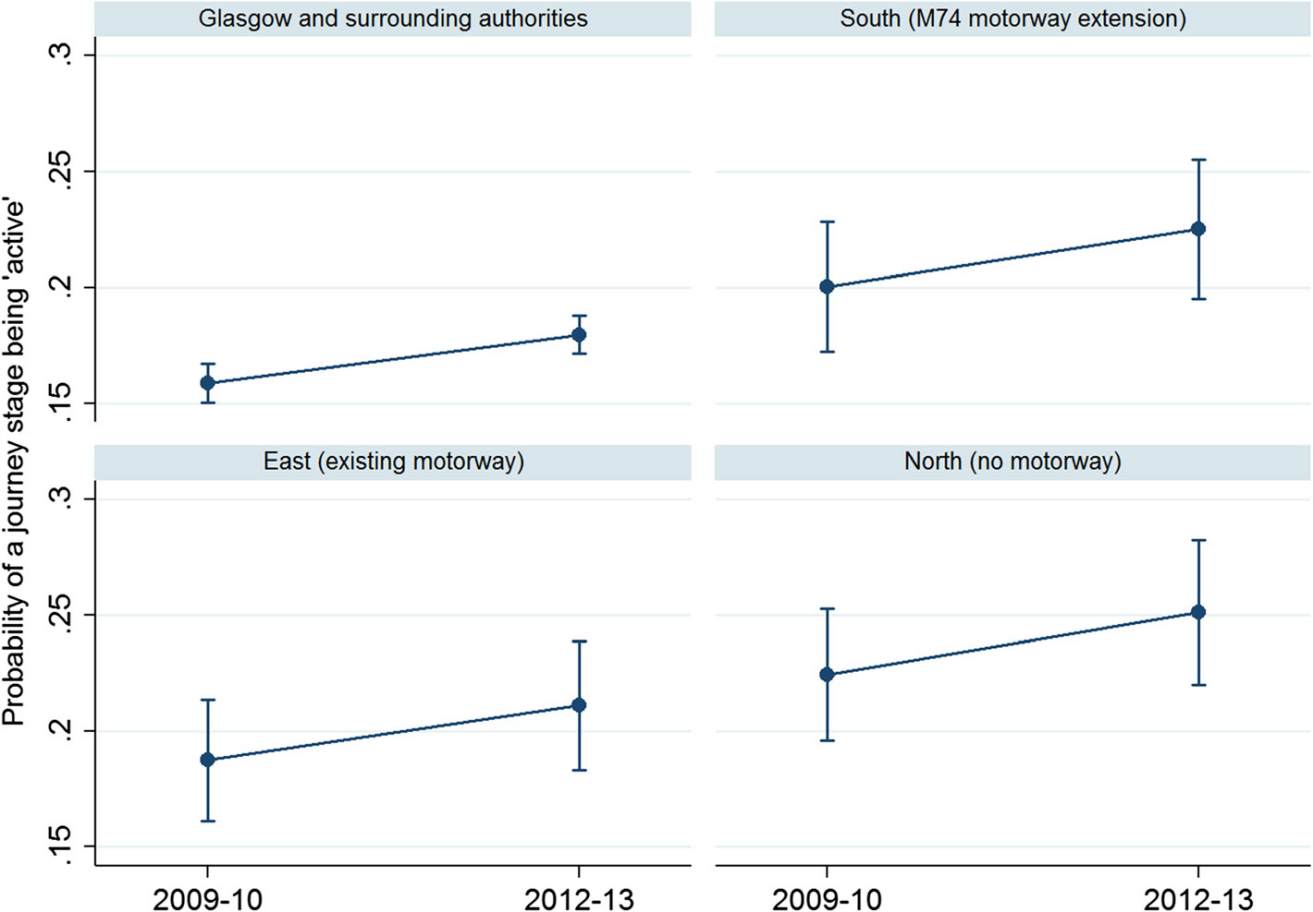
Predictive margins of probability of journey being ‘active’ by study area

The probability of a stage being made ‘actively’ increased in all study areas (Fig. 2). Although these graphs display wide confidence intervals, they show that there were increases in actively travelled journeys and that these increases were of a similar magnitude across all three study areas, and Glasgow and surrounding authorities. The proportion of active stages is shown in Table 2.

**Table 2:** Proportion of journey stages using active travel

Proportion active travel	Glasgow and surrounding authorities	Study Area 1 (South)	Study Area 2 (East)	Study Area 3 (North)
2009/10	18.1 (n:1766)	21.8 (n:95)	21.4 (n:102)	20.9 (n:113)
2012/13	19.8 (n:2309)	23.6 (n:128)	24.6 (n:138)	22.9 (n:136)

Includes all journey stages regardless of length

### Comparing changes in active travel over time between areas

#### Likelihood of journey stages using active travel 2009–10 to 2012–13

The likelihood of an active stage was modelled for the periods 2009–10 and 2012–13 for each study area against the reference category Glasgow and surrounding authorities (Table 3). There was little difference in the odds ratio (OR) of an active stage compared to Glasgow and surrounding authorities for the South (intervention) and East (comparator area) by time-period both in the unadjusted and adjusted models (adjusted for age, gender, employment and health status). The North study area, containing no motorway, showed significantly higher active stages compared to Glasgow and surrounding authorities in both the adjusted and unadjusted models for the more recent time period (OR:1.79, *p* = 0.003).

**Table 3:** Likelihood of journey stage using active travel methods (weighted)

	Unadjusted							
	2009/10				2012/13			
	OR	*p*	LL 95 % CI	UL 95 % CI	OR	*p*	LL 95 % CI	UL 95 % CI
Glasgow and surrounding authorities	REF							
Study Area 1 (South)	1.26	0.209	0.88	1.80	1.37	0.081	0.96	1.96
Study Area 2 (East)	1.39	0.078	0.96	2.02	1.24	0.240	0.87	1.77
Study Area 3 (North)	1.26	0.228	0.86	1.85	1.77	0.003	1.22	2.56
	Adjusted~							
	2009/10				2012/13			
	OR	*p*	LL 95 % CI	UL 95 % CI	OR	*p*	LL 95 % CI	UL 95 % CI
Glasgow and surrounding authorities	REF							
Study Area 1 (South)	1.29	0.193	0.88	1.88	1.37	0.092	0.95	1.99
Study Area 2 (East)	1.38	0.108	0.93	2.05	1.13	0.513	0.79	1.62
Study Area 3 (North)	1.33	0.136	0.91	1.95	1.79	0.003	1.22	2.60

Adjusted for age, gender, employment and health status

OR: Odds ratio

LL 95 % CI: Lower level 95 % confidence interval. UL 95 % CI: Upper level 95 % confidence interval

When the analyses were repeated to examine whether stage length (i.e. if the stage could plausibly be made using active travel) influenced the likelihood of an active stage, the sensitivity analysis provided no changes in the results (Additional file 2: Table S1). Similarly, no between study area differences were found using an alternative reference category of ‘Glasgow City’.

Interactions of time-period and study area as predictors of active travel showed that the South (intervention) and North (Control) had an increased odd ratio of an active stage during 2012–13 when compared to Glasgow and its surrounding authorities in 2009/10 (Table 4).

**Table 4:** Interaction of between study area differences in change of using active travel over time (weighted)

	2009/10				2012/13			
	OR	*p*	LL 95 % CI	UL 95 % CI	OR	*p*	LL 95 % CI	UL 95 % CI
Glasgow and surrounding authorities	REF				1.16	0.076	0.99	1.36
Study Area 1 (South)	1.29	0.181	0.89	1.89	1.6	0.015	1.11	2.34
Study Area 2 (East)	1.38	0.108	0.93	2.05	1.32	0.146	0.91	1.91
Study Area 3 (North)	1.34	0.133	0.91	1.95	2.07	<0.001	1.4	3.05

Chi2(7): 21.02, p:0.0037

Interaction performed: Assessment of change in the likelihood of an active journey stage over time and between study area based on reference category of Glasgow and surrounding authorities in 2009/10

Adjusted for age, gender, employment and health status

OR: Odds ratio

LL 95 % CI: Lower level 95 % confidence interval. UL 95 % CI: Upper level 95 % confidence interval

## Discussion

### Summary

The aim of our study was to describe (a) recent changes in active travel over time and, (b) whether the opening of the M74 extension in Glasgow produced any between area differences in change compared to journeys completed in a comparator, control and wider city area. We found increases in the proportion of journey stages made using active travel from 2009–10 to 2012–13 for all study areas and both Glasgow and surrounding local authorities, and Glasgow City. There was no significant change in the likelihood of a journey stage being ‘active’ for people living near the M74 extension compared to Glasgow and surrounding authorities, and in a comparator area. There were small increases in the proportion of stages using active travel and the probability of a stage being made actively.

Therefore in evaluating whether the opening of the M74 extension had an effect on active travel in the local area: we found no clear evidence that the M74 extension alone had either increased or decreased actively travelled journey stages in the local area. Increases in active travel were more likely due to changes in active transport for the region as a whole.

### Comparison with existing literature

The potential effects of the M74 extension on local residents were considered prior to its construction [16] and described in terms of two ‘extreme case’ vignettes; a virtuous and vicious spiral. In relation to active travel the virtuous cycle proposed that the new motorway extension could lead to reduced traffic on local roads; making conditions more pleasant for pedestrians and cyclists, and encouraging opportunities for physical activity to increase. Alternatively, the vicious spiral hypothesised increased traffic on local roads may encourage people to travel both further and by car, that the motorway and junctions would degrade the local environment making it less pleasant or safe for cyclists and pedestrians, and that opportunities for physical activity as well as well-being of local people would decline. The active travel data alone suggest that neither of the two hypothesised spirals of extreme effects have been realised. Indeed, our results showed that there was no indication of change either way.

Cross-sectional surveys conducted in three areas of the UK (Cardiff, Kenilworth and Southampton) prior to new walking and cycling infrastructure described that for obligatory journeys people tended to choose the fastest mode of transport available to them [18]. This is often because motorised transportation and long-distance journeys are negatively associated with active travel [18]. While the M74 extension did have potential to reduce journey times by moving traffic onto a quicker free flowing motorway and increase journeys made by car, this has not produced an immediate negative impact on actively travelled journey stages made by local residents. However, our analysis did not consider the city-wide impact of the motorway on active journey stages by comparing Glasgow to other urban towns and cities in Scotland. This is currently underway.

A stimulating built environment around the home containing interesting things to look at increases the likelihood of physical activity [19]. It is doubtful whether the M74 extension, which is overhead and imposing in places, fits this definition. Nevertheless, journey stages completed actively in the area did not decline but remained stable with small, albeit non-significant, increases in the proportion of active journey stages. These increases were consistent with other areas of the city when compared to Glasgow and surrounding authorities.

The results presented in our study showed increases in stages being made actively from the period 2009–10 to 2012–13 in Glasgow and surrounding authorities, and all three of our study areas. In England between the periods 2012–13 to 2013–14 there were no countrywide increases in cycling and walking, however there were regional differences in uptake and 35 local authorities reported significant increases in the proportion of local people who cycled one or more times a week [20]. The England and Wales Census in 2011 showed that for the first time participation in cycling and walking had stabilized rather than continuing a downward trend, and that both cycling and using public transport showed small increases in participation [21].

In encouraging people to build physical activity into their daily lives it has been suggested that tackling environmental, structural and financial barriers to active travel should be prioritised [22]. In the area surrounding the new M74 extension there were no specific investment or promotion interventions which aimed to increase active travel. This could be considered as the key explanatory factor as to why the new M74 extension did not produce any increase in journey stages made actively by people living near the M74 extension compared to people living near existing transport structures and the wider city region.

New urban motorway infrastructure in the UK is rare; therefore both the anticipated and true impact of the M74 extension on active travel is unknown; arguments made during the consultation phase lacked a clear evidence base. Construction provided a unique opportunity to conduct a natural experiment to understand the impact of the M74 extension on active travel behaviours of local residents. This evidence will have significant importance for future urban transport developments both in the UK and other nations world-wide.

### Strengths and weaknesses

There are important strengths and weaknesses in the study design which should be considered when drawing final conclusions. The SHS travel diaries are part of an on-going repeat cross sectional survey which includes a large randomly selected and representative population. To describe between area differences in active journey stages between areas of Glasgow and surrounding authorities, individuals were assigned to one of three study areas based upon whether their residential intermediate zone was fully or partially located within a study area. This method is not as accurate as assigning a person to an area using a full postcode as sometimes these intermediate zones cover large geographic areas. Due to data protection concerns, the data we obtained did not include precise postcode locations to allow matching [16]. The main analyses were also limited in terms of final sample size and consequent statistical power.

The SHS travel diary dataset collects information for all stages of a journey undertaken between the ultimate start and end destination of a journey. This allowed us to analyse stage data and increase the final numbers included in our analysis. The SHS calculated journey distances using straight line linear distance between two postcode points. This may under report the true distance of a journey where routes cannot follow this path and are usually longer.

The likelihood of a stage being made either by foot or cycled may largely depend upon the distance travelled and longer stage distances are negatively associated with active travel [18]. A recent study included only stages that had a ‘*reasonable transport mode choice*’ defined as over 0.5 km and under 5 km when measuring the likelihood of active travel [23]. This assumed that most people will ordinarily walk or cycle a journey under 0.5 km in distance and, unless a cycling or walking enthusiast, would not ordinarily walk or cycle a distance over 5 km. The analysis presented in the main body of our paper did not include any stage distance parameters but within our sensitivity analysis we applied a number of different parameters. When applying these parameters they had little impact on the overall messages and results of the analysis.

Although there are limitations when using national datasets such as the SHS travel diaries, our study in part shows the potential and benefit of using routinely collected data. The dataset we analysed included almost 8000 people completing 21461 journey stages over two time periods across Glasgow and surrounding authorities. Using these data for research purposes incurred no charges and compared to collecting our own study data for this number of people it would have been both burdensome and expensive.

## Conclusions

Glasgow, the largest city in Scotland, displayed small increases in the proportion of journey stages which were walked or cycled but there were no between area differences in change in active travel for people living near the new M74 extension when compared to Glasgow and surrounding authorities. It must be noted that although there was not a significant increase in active travel journeys made by local residents, neither was there a decrease.

Although we found little city-wide variation in active travel, recent studies have suggested that there is regional variation in increases in active travel. Future studies exploring regional variation and inequalities in active travel could provide more understanding of whether changes in active travel in Glasgow are comparable to other UK cities and large urban areas.

The M74 extension is here to stay, therefore it is now important that transport activity and active travel monitoring continues in the area. The Scottish Government maintains this at a national level through the National Indication: Public or Active transport, which aims to ‘Increase the proportion of journeys to work made by public or active transport’ [24] as captured using SHS data. It is important that analyses of these data, and transport activity, are conducted at a local level to provide insights of changes to the local environment which could potentially encourage or discourage physical activity.

## Abbreviations

CI, confidence interval; KM, kilometre; LL, lower level; N, Number; NHS, National Health Service; NICE, National Institute for Health and Clinical Excellence; OR, odds ratio; SHS, Scottish Household Survey; UK, United Kingdom; UL, upper level

## Additional files

Additional file 1: Table S2. Description of Dichotomised variables from SHS. (XLSX 11 kb) Additional file 2: Table S1. Sensitivity analysis of RR of journey stages being made by active means. (XLSX 13 kb)

## References

[1] Take action on active travel [http://www.fph.org.uk/uploads/Take_action_on_active_travel.pdf]. Accessed 13 Dec 2015.

[2] Götschi T, Tainio M, Maizlish N, Schwanen T, Goodman A, Woodcock J (2015). Contrasts in active transport behaviour across four countries: How do they translate into public health benefits?. Preventive medicine.

[3] Jarrett J, Woodcock J, Griffiths UK, Chalabi Z, Edwards P, Roberts I, Haines A (2012). Effect of increasing active travel in urban England and Wales on costs to the National Health Service. The Lancet.

[4] Woodcock J, Edwards P, Tonne C, Armstrong BG, Ashiru O, Banister D, Beevers S, Chalabi Z, Chowdhury Z, Cohen A (2009). Public health benefits of strategies to reduce greenhouse-gas emissions: urban land transport. The Lancet.

[5] Ferdinand AO, Sen B, Rahurkar S, Engler S, Menachemi N (2012). The relationship between built environments and physical activity: a systematic review. Am J Public Health.

[6] Excellence NIfHaC: Guideline [PH8] Physical activity and the environment London; 2008.

[7] Excellence NIfHaC: Guideance update 57: Physical activity and the environment. London; 2014.

[8] Parkin J: Cycling and sustainability. Emerald Group Publishing; 2012.

[9] Badland HM, Oliver M, Kearns RA, Mavoa S, Witten K, Duncan MJ, Batty GD (2012). Association of neighbourhood residence and preferences with the built environment, work-related travel behaviours, and health implications for employed adults: Findings from the URBAN study. Social Science & Medicine.

[10] Ogilvie D, Mitchell R, Mutrie N, Petticrew M, Platt S (2008). Personal and environmental correlates of active travel and physical activity in a deprived urban population. International Journal of Behavioral Nutrition and Physical Activity.

[11] Glasgow City Council Area - Demographic Factsheet [http://www.nrscotland.gov.uk/files/statistics/council-area-data-sheets/glasgow-city-factsheet.pdf]. Accessed 22 Jan 2016.

[12] Hickman R, Watt D: Roads (Scotland) Act 1984: Acquisition of Land (Authorisation Procedure)(Scotland) Act 1947: M74 Special Road (Fullarton Road to West of Kingston Bridge) Orders: Report of Public Local Inquiry Into Objections. Scottish Executive; 2005.

[13] Management ER: The M74 Completion: Environmental Statement (Executive S ed., vol. 1; 2003.

[14] Scottish Household Survey [http://www.gov.scot/Topics/Statistics/16002]. Accessed 13 Dec 2016.

[15] Scottish Neighbourhood Statistics: Intermediate Geography Background Information [http://www.gov.scot/Publications/2005/02/20732/53083]. Accessed 13 Dec 2015.

[16] Ogilvie D, Mitchell R, Mutrie N, Petticrew M, Platt S (2006). Evaluating health effects of transport interventions: methodologic case study. Am J Prev Med.

[17] Scotland's Census 2011 [http://www.scotlandscensus.gov.uk/]. Accessed 13 Dec 2015.

[18] Song Y, Preston JM, Brand C (2013). What explains active travel behaviour? Evidence from case studies in the UK. Environment and Planning A.

[19] Adlakha D, Hipp AJ, Marx C, Yang L, Tabak R, Dodson EA, Brownson RC (2015). Home and workplace built environment supports for physical activity. American journal of preventive medicine.

[20] Local Area Walking and Cycling Statistics: England, 2013/14 [https://www.gov.uk/government/uploads/system/uploads/attachment_data/file/437001/local-area-walking-and-cycling-statistics-england-2013-14.pdf]. Accessed 22 Feb 2016.

[21] Goodman A (2013). Walking, cycling and driving to work in the English and Welsh 2011 census: trends, socio-economic patterning and relevance to travel behaviour in general. PloS one.

[22] Laverty AA, de Sá TH, Monteiro CA, Millett C (2015). Getting sedentary people moving through active travel. BMJ.

[23] Rind E, Shortt N, Mitchell R, Richardson EA, Pearce J (2015). Are income-related differences in active travel associated with physical environmental characteristics? A multi-level ecological approach. International Journal of Behavioral Nutrition and Physical Activity.

[24] National Indicators: Proportion of adults usually travelling to work by public or active transport [http://www.gov.scot/About/Performance/scotPerforms/indicator/transport]. Accessed 22 Feb 2016.

